# Comparative transcriptome analysis of flower heterosis in two soybean F1 hybrids by RNA-seq

**DOI:** 10.1371/journal.pone.0181061

**Published:** 2017-07-14

**Authors:** Chunbao Zhang, Chunjing Lin, Fuyou Fu, Xiaofang Zhong, Bao Peng, Hao Yan, Jingyong Zhang, Weilong Zhang, Pengnian Wang, Xiaoyang Ding, Wei Zhang, Limei Zhao

**Affiliations:** 1 Soybean Research Institute, National Engineering Research Center for Soybean, Jilin Academy of Agricultural Sciences, Changchun, China; 2 Department of Botany and Plant Pathology, Purdue University, West Lafayette, United States of America; 3 Agro-Biotechnology Institute, Jilin Academy of Agricultural Sciences, Changchun, China; Dokuz Eylul Universitesi, TURKEY

## Abstract

Heterosis has been widely exploited as an approach to enhance crop traits during breeding. However, its underlying molecular genetic mechanisms remain unclear. Recent advances in RNA sequencing technology (RNA-seq) have provided an opportunity to conduct transcriptome profiling for heterosis studies. We used RNA-seq to analyze the flower transcriptomes of two F1 hybrid soybeans (HYBSOY-1 and HYBSOY-5) and their parents. More than 385 million high-quality reads were generated and aligned against the soybean reference genome. A total of 681 and 899 genes were identified as being differentially expressed between HYBSOY-1 and HYBSOY-5 and their parents, respectively. These differentially expressed genes (DEGs) were categorized into four major expression categories with 12 expression patterns. Furthermore, gene ontology (GO) term analysis showed that the DEGs were enriched in the categories metabolic process and catalytic activity, while Kyoto Encyclopedia of Genes and Genomes (KEGG) pathway enrichment analysis found that metabolic pathway and biosynthesis of secondary metabolites were enriched in the two F1 hybrids. Comparing the DEGs of the two F1 hybrids by GO term and KEGG pathway analyses identified 26 common DEGs that showed transgressive up-regulation, and which could be considered potential candidate genes for heterosis in soybean F1 hybrids. This identification of an extensive transcriptome dataset gives a comprehensive overview of the flower transcriptomes in two F1 hybrids, and provides useful information for soybean hybrid breeding. These findings lay the foundation for future studies on molecular mechanisms underlying soybean heterosis.

## Introduction

Heterosis has been widely used for the increase and exhibition of superior phenotypes in crop breeding, such as enhanced biomass production, development rate, grain yield, and stress tolerance. Hybrid rice, which occupies more than 50% of the total rice growing area in China, has a 10%–20% yield advantage over inbred varieties [1]. However, little is known about the molecular genetic mechanisms of heterosis.

Dominance [2, 3] and over-dominance [4] were two hypotheses considered in the early 20^th^ century to explain heterosis. Moreover, nonadditive behavior was described as the consequence of genetic differences between distinct homozygous parental lines and their heterozygous hybrids [5]. With the development of functional genomics, large-scale transcriptome analysis has been used to investigate heterosis in *Arabidopsis* [6, 7], maize [8], and rice [9–11]. These studies partially unveiled the molecular basis of heterosis at the transcriptional level [12, 13]. Since then, next-generation high-throughput RNA sequencing (RNA-seq) has been developed to discover, profile, and quantify RNA transcripts [14, 15]. It has also been used to study the mechanisms of heterosis in interspecific F1 triploids or F1 hybrids of cotton [16], wheat [17], and rice [18, 19], but few studies have investigated heterosis in soybean using RNA-seq.

Soybean (*Glycine max* (L.) Merr) is an important crop that provides plant protein and oil. However, its low yield has restricted soybean development over past decades. Hybrid soybeans are known to demonstrate heterosis, similar to maize, rice, and oilseed [20]. Davis had identified soybean cytoplasmic male sterility (CMS) in his United States patent [21], but other breeders and researchers did not continue to study this because they were unable to replicate his experiments. More recently, male sterile F1 soybean plants caused by both translocation and cytoplasmic–nuclear interactions were reported by crossing *G*. *max* accession 167 and *Glycine soja* accession 035 [22].

A CMS soybean line (OA) and maintainer line (OB) were developed through repeated backcrossing with wild soybean line 035, with CMS soybean line OA retaining the typical wild soybean ecotype of its parent. Because RN-type which derived from Ru Nan Tian E Dan male-sterile cytoplasms, were used for initial hybrid seed production in *G*. *max*, almost all soybean hybrids possess RN genotypes. As of 2013, over 200 pairs of stable sterile and maintainer lines have been bred from RN-type male-sterile cytoplasm. The first soybean CMS three-line system, comprising a male-sterile line, a maintainer line, and a restorer line, was developed by Sun et al in 1994 [23]. This achievement showed that it is possible to utilize soybean heterosis for hybrid soybean breeding. Sun et al later applied the USA pattern in 2001 [24], and HYBSOY-1 HYBSOY-1 was the first commercialized hybrid soybean variety to be released in the world in 2002. HYBSOY-1 not only has a high yield but also good resistance to disease and a high seed quality [25]. Since 2002, 16 commercialized hybrid soybean varieties have been released.

Previous studies have demonstrated that heterosis levels might be higher in root traits than in above-ground agronomic traits [19, 26–28]. The plant flower is a crucial organ which serves a number of important functions, including the generation of germ cells, insemination, seed formation, amino acid production, the facilitation of metabolic pathways, and hormone production. Because these traits are all directly related to plant seed products, the flower is an ideal organ for investigating the genetic basis of soybean seed heterosis, although this has not yet been done systematically.

In this study, we focused our heterosis research on two F1 soybean hybrids varieties, HYBSOY-1 and HYBSOY-5. These were developed by the soybean CMS three-line system, using a restorer line crossed with different two male-sterile lines. We used RNA-seq to investigate the global transcriptomes of flowers from HYBSOY-1 and HYBSOY-5 and their parents. Differentially expressed transcripts were analyzed between parent and offspring plants, and their expression patterns were determined to identify potential candidate genes responsible for heterosis. Several candidate genes were found to be involved in the categories metabolic process and catalytic activity. We expect this genome-wide transcriptome comparison to provide a starting point for understanding the causative mechanism of altered gene expression in hybrids and the molecular mechanisms underlying soybean heterosis.

## Materials and methods

### Plant material and growth conditions

The two F1 hybrid soybean varieties HYBSOY-1 and HYBSOY-5 and their parents JLCMS9A (male), JLCMS84A (male), and JLH1 (female) were used in this study. All plants were planted in a randomized block design of three replications, with a length of 5 m and a width of 65 cm for each row, and a space of 15 cm between each plant at the Jilin Academy of Agricultural Sciences, China in 2013. The mix flowers were collected from twelve plants every genotype and stored at -80°C in preparation for RNA-Seq analysis.

### Soybean seed heterosis measurements

Agronomic traits were investigated over 2 years with three replications. The protein content (PC, %) and oil content (OC, %) were measured by the Perten DA7200 NIR Analyzer (Sweden) using 50 g samples of each plant. Other measurements were pods per stem (NPS), indicating the number of pods with normal seeds; the 100 seeds weight (HSW; g), indicating the weight of 100 normal seeds of each plant; plant height (PH; cm), indicating the length from the cotyledonary node to the top of the plant; nodes of the main stem (NNS), indicating the number of nodes from the cotyledonary node to the top of the main stem; and number of seeds per plant (NSP), indicating the number of normal seeds per plant. The average of 10 plants was used for these measurements with three replications.

Mid-parent heterosis (MPH) and best-parent heterosis (HPH) were calculated according to the following formulae: MPH = ((F_1_-MP)/MP)×100% and HPH = ((F_1_-HP)/HP)×100%, where F1 is the traits of the hybrids, MP is the average traits of two parents, and HP is the best trait of two parents. Hypothesis testing was performed using the *t*-test.

### Total RNA extraction, cDNA library construction, and Ion Porton deep sequencing

Total RNA was extracted from each sample using TRIzol Reagent (Life Technologies, USA) according to the manufacturer’s protocol. The concentration of each sample was measured by a NanoDrop 2000 spectrophotometer (Thermo Scientific, USA), and the quality was assessed by the Agilent 2200 TapeStation system (Agilent, USA). A sequencing library for each RNA sample was prepared using the Ion Total RNA-Seq Kit v2 according to the manufacturer’s protocol (Life Technologies). Briefly, poly (A)-containing mRNA was purified from 5 μg total RNA using Dynabeads (Life Technologies). mRNA was fragmented using RNase III and purified, then hybridized and ligated with an Ion adaptor. The RNA fragments were reverse-transcribed and amplified into double-stranded cDNA. This was then purified using magnetic beads, and the molar concentration was determined for each cDNA library. Emulsion PCR was performed using the cDNA library as a template. Template-positive Ion PITM Ion Sphere™ Particles were enriched and loaded onto the Ion PITM chip for sequencing.

### Data analysis of RNA-Seq

Raw data (raw reads) in FASTQ format were first processed through in-house perl scripts. In this step, clean data (clean reads) were obtained by removing reads containing adapters or poly-N, and low-quality reads. At the same time, Q20, Q30, and the GC content of clean data were calculated. All downstream analyses were based on high-quality clean data. Reference genome and gene model annotation files were downloaded directly from the genome website (http://phytozome.jgi.doe.gov/pz/portal.html#!info?alias=Org_Gmax). A reference genome index was built using Bowtie v2.2.3 [29] and paired-end clean reads were aligned to the reference genome using TopHat v2.0.12 [30]. We selected TopHat as the mapping tool because it can generate databases of splice junctions based on the gene model annotation file, therefore achieving a better mapping result than other non-splice mapping tools. HTSeq v0.6.1 was used to count the number of reads mapped to each gene [31]. The fragments per kilobase of transcript per million base pairs sequenced (FPKM) was then calculated for each gene based on the length of the gene and reads counts mapped to this gene. FPKM simultaneously considers the effect of sequencing depth and gene length for the reads count, and is currently the most commonly used method for estimating gene expression levels. RNA-Seq quality was evaluated using Pearson’s correlation coefficient among samples.

### Quantitative real-time PCR validation of transcriptome data

To validate the transcriptome data, the expression of 20 genes was evaluated by quantitative real-time PCR (qRT-PCR) analysis. cDNAs were synthesized according to the manufacturer’s protocol (Takara, Dalian, China) and used as template for qRT-PCR analysis using primers based on the reference soybean gene sequences (S1 Table). qRT-PCR was conducted using UltraSYBR mixture (CWBIO, China) in a typical 20μl PCR mixture that included 10μl of UltraSYBR mixture, 2μl (100 ng) of template cDNA, and 0.4μM of each PCR primer. Cycling conditions were 95°C for 2 min, followed by 40 cycles of 95°C for 10 s (denaturation), followed by 60°C for 20 s (annealing and extension). The melting curve of each PCR amplicon was obtained under the following conditions: 95°C for 10 s followed by a constant increase in temperature from 65 to 95°C at an increment of 0.5°C / cycle. Samples were run on the StepOnePlus Real-Time PCR System (ABI, USA). Relative expression of the target genes was analyzed with the 2^-ΔΔCt^ method using ABCT,CONS4,ACT11 as internal controls [32]. All samples were amplified with three biological replications and with three technical replication each biological replication.

### Differential expression analysis

Differential expression analysis of two conditions was performed using the DEGSeq R package (1.20.0) [33]. *P* values were adjusted using the Benjamini–Hochberg method. Corrected *P*-values of 0.005 and a log_2_ (fold-change) of 1 were set as the threshold for significant differential expression. Gene Ontology (GO) enrichment analysis of differentially expressed genes (DEGs) was implemented by the GOseq R package [34], in which gene length bias was corrected. GO terms with corrected *P*-values <0.05 were considered significantly enriched by DEGs.

KEGG is a database resource that uses information at the molecular level, especially large-scale molecular datasets generated by genome sequencing and other high-throughput experimental technologies, to understand high-level functions and utilities of the biological system [35] such as the cell, the organism, and the ecosystem (http://www.genome.jp/kegg/). We used KOBAS software [36] to test the statistical enrichment of DEGs in KEGG pathways.

## Results

### Production and phenotypes of F1 hybrids

**Fig 1:**
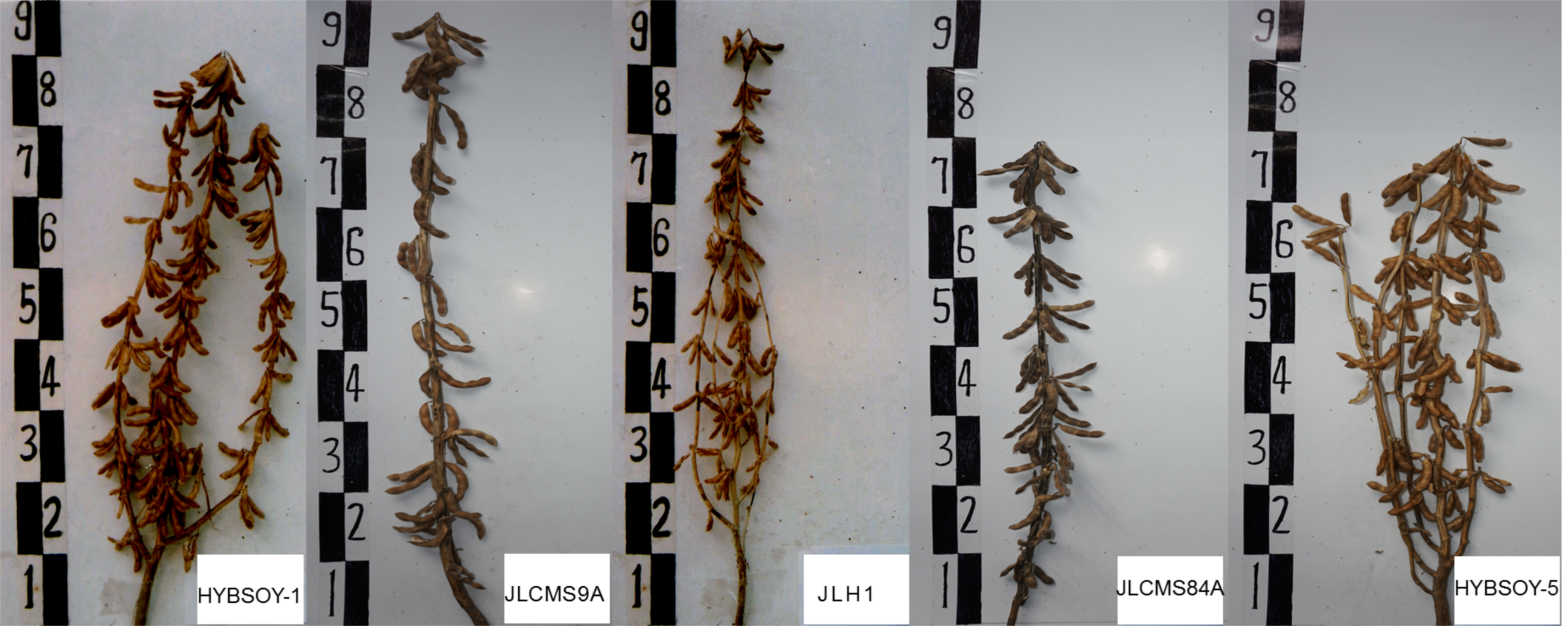
The phenotype of F1 hybrids (HYBSOY-1 and HYBSOY-5) and their parents (JLCMS9A, JLCNS84A, and JLH1).

Two F1 hybrid plants (HYBSOY-1 and HYBSOY-5) were produced by crossing JLCMS9A and JLCMS84A with JLH1. JLCMS84A and JLCMS9A are two male-sterile lines from an RN-type male-sterile cytoplasm. HYBSOY-1 and HYBSOY-5 have been released as hybrid varieties in China and are widely grown throughout northeastern China because of their important heterosis. In this study, heterosis of agronomic traits and seed quality traits was detected, including PH, NNS, NPS, NSP, HSW, and PC and OC. We did not detect significant heterosis in PH, PC, or OC; however, significant heterosis was identified in four agronomic traits: NNS, NPS, NSP, and HSW (Fig 1 and Table 1).

**Table 1:** Comparison of agronomic traits for HYBSOY-1 and HYBSOY-5 with their parents (x ± SD).

	Plant height (cm)	Number of nodes per stem	Number of pods per plant	Number of seeds per plants	100-seed weight (g)	Protein content (%)	Oil content (%)
JLCMS9A	99.1±3.9^a^	15.4±1.3^d^	38.2±5.4^c^	91.5±8.8^d^	20.3±0.6^a^	39.53±0.3^a^	19.93±0.2^d^
JLH1	100.6±2.8^a^	22.8±2.0^b^	56.3±8.7^b^	107.0±8.1^c^	15.5±0.4^c^	38.29±0.2^b^	22.22±0.3^ab^
HYBSOY-1	94.7±2.9^b^	22.8±1.4^b^	63.3±10.4^a^	127.1±10.0^b^	19.0±0.6^b^	39.39±0.4^a^	21.48±0.4^bc^
MPH (%)	-5.2	19.37	33.97	28.06	6.15	1.23	1.92
HPH (%)	-4.4	0	12.43	18.79	22.58	2.87	-3.33
JLCMS84A	67.0±2.9^c^	17.1±1.3^c^	34.9±4.7^c^	77.8±9.0^e^	14.7±0.8^c^	37.29±0.2^c^	20.69±0.2^cd^
HYBSOY-5	67.3±2.5^c^	25.9±1.2^a^	65.4±10.6^a^	145.3±11.1^a^	18.6±0.8^b^	38.79±0.3^ab^	22.38±0.3^a^
MPH (%)	-19.7	29.82	43.42	57.25	23.18	2.65	4.31
HPH (%)	0.44	13.60	16.16	35.80	20	1.31	0.72

MPH = (F1-MP)/MP×100%, MP = (P1+P2)/2; HPH = (F1-HP)/HP×100%. F1 value represents the F1 hybrid trait. MP value shows the mean trait value of two parents. HP indicates the trait value of the higher parent. Lowercase letters within the same column show significant difference at the 0.05 level.

MPH and HPH were calculated to measure the heterosis of HYBSOY-1 and HYBSOY-5. We observed significant MPH (*P*<0.05) for NNS, NPS, NSP, and HSW in both HYBSOY-1 and HYBSOY-5. Furthermore, significant HPH of these traits was also observed in both F1 hybrids. The degree of heterosis for these traits was greater in HYBSOY-5 than in HYBSOY-1, with the MPH ranging from 23.18% to 57.25%.

### Genome-wide gene expression level divergence between parents and F1 hybrids

A total of 385,110,464 high quality RNA-seq reads were generated using a Life Technologies Ion Proton sequencer (Table 2). After filtering and trimming the adaptors and low-quality reads, 315,385,716 high-quality reads were obtained which were mapped to the soybean reference genome using TopHat v2.0.12. The ratio of alignment was 76.99%–82.83% (Table 2). Finally, we used the Pearson’s correlation coefficient among samples to evaluate the difference among of samples (S1 Fig, S1 and S2 Tables).

**Table 2:** Alignment results of RNA-seq data.

Libraries		Total read	Mapped reads	Percentage of mapped reads
**JLCMS9A**	M1	70,304,473	59,507,471	77%
**HYBSOY-1**	F1	85,318,220	70,657,223	83%
**JLH1**	P	65,268,445	52,394,816	80%
**JLCMS84A**	M2	87,695,030	71,282,039	81%
**HYBSOY-5**	F1	76,524,296	61,544,167	80%

**Fig 2:**
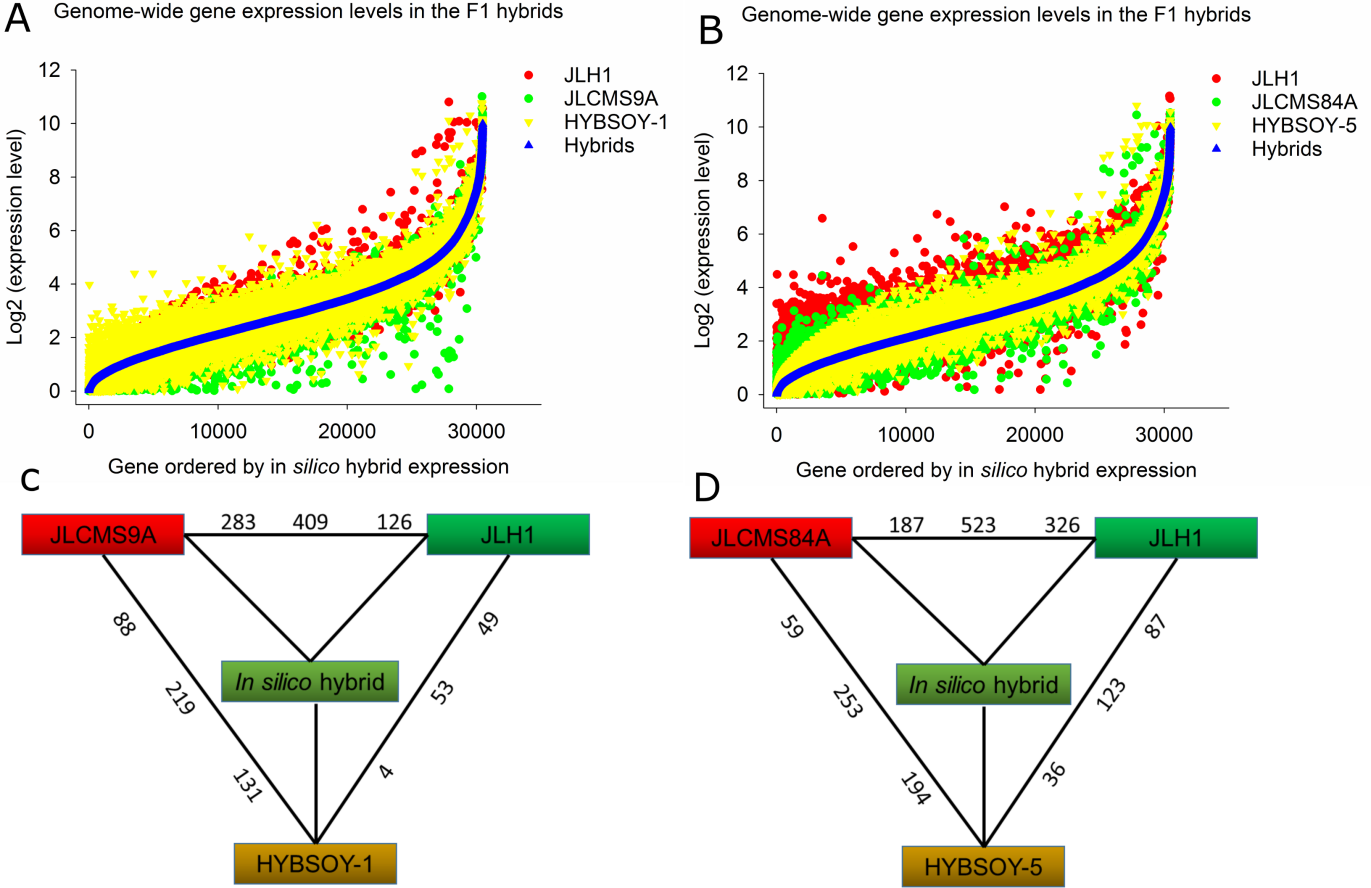
Transcriptome profiling and differentially expressed genes between F1 hybrids and their parents. **(A)** and **(B)** Genome-wide gene expression in F1 hybrids, the *in silico* hybrids, and the two parents. **(A)** F1 hybrid HYBSOY-1 and two parents (JLCMS9A and JLH1). **(B)** F1 hybrid HYBSOY-5 and two parents (JLCMS84A and JLH1). **(C)** and **(D)** Number of differentially expressed genes of pair-wise comparisons of all materials. Black number indicates the total number and proportion of genes that are differentially expressed in each comparison. Also shown for each contrast is the partitioning of the total number of differentially expressed genes into the direction of upregulation. For example, in C, 409 genes are indicated as being differentially expressed between JLCMS9A and JLH1. Of these, 283 genes are upregulated in JLCMS9A, and 126 genes are upregulated in JLH1.

Based on the substantial phenotypic disparity among the three parents and the discernible novel phenotypes exhibited by their F1 hybrids, we next explored the extent of transcriptome divergence between parents and offspring to explain its possible association with heterosis in soybean. First, we performed pair-wise comparisons between parents to assess pre-existing differential gene expression (Fig 2A and 2B). To accurately compare the gene expression between parents and hybrids, we constructed independent *in silico* hybrids by mixing the RNA-seq data of two pairs of sequenced parental individuals. Many DEGs were identified between the two parents (Fig 2A and 2B, non-overlapping blue and red dots). The expression level of most F1 hybrid genes overlapped with that of the *in silico* hybrids (black curve). However, a substantial proportion of F1 hybrid genes showed higher or lower expression levels than those of *in silico* hybrids or their parents (Fig 2A and 2B, green dots).

We next carried out pair-wise comparisons of the two F1 hybrids and their parents using the algorithm DEGseq [37]. DEG analysis between F1 hybrids and their parents, and between F1 hybrids and *in silico* hybrids, removed those genes with additive expression from F1 hybrids expected by the null hypothesis. This showed that a total of 681 and 899 genes had nonadditive expression in HYBSOY-1 and HYBSOY-5, respectively, with more than two-fold changes (*P*<0.05 with false discovery rate (FDR)<0.05) between F1 hybrids and parents (Fig 2C and 2D).

**Fig 3:**
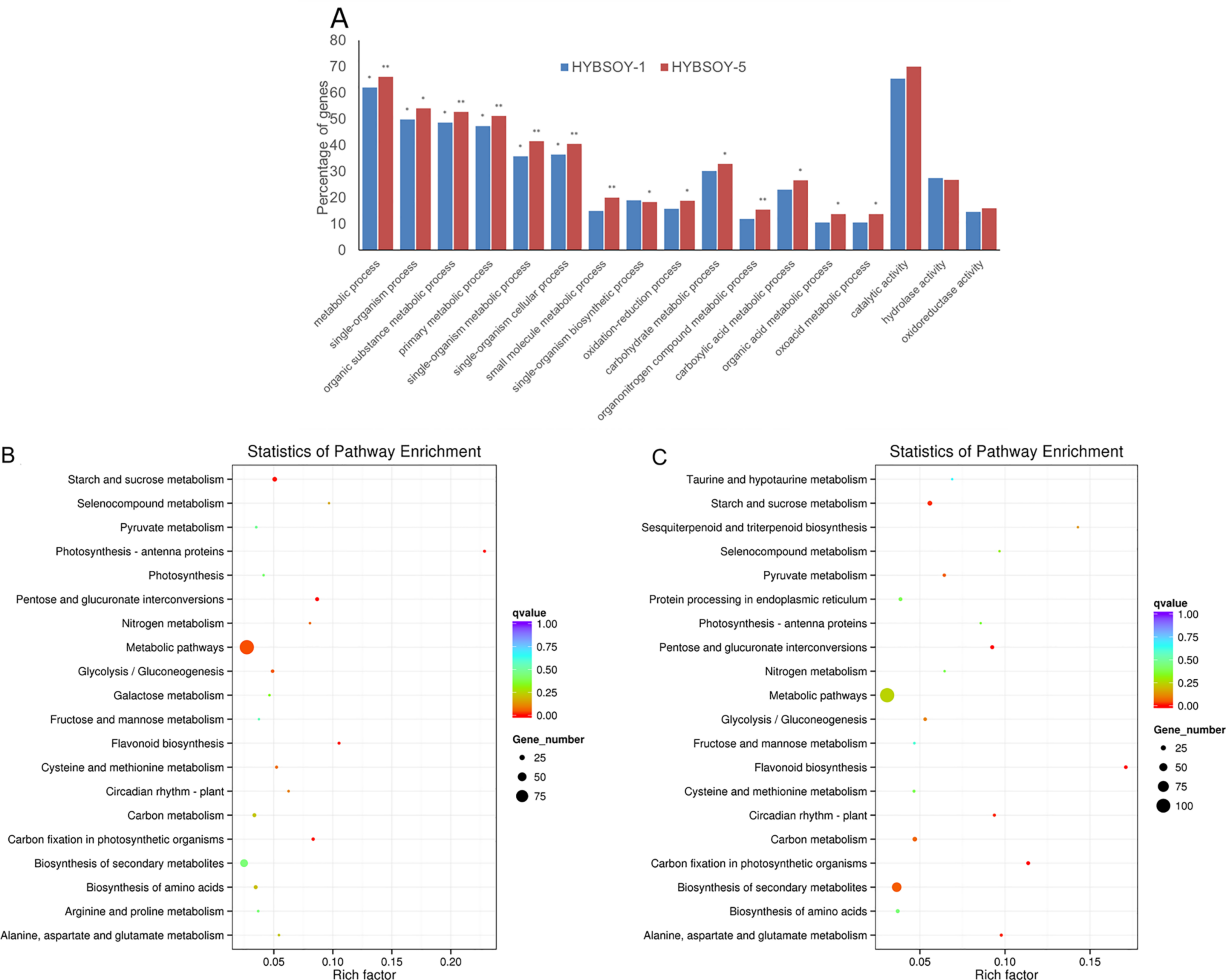
Differential gene expression in soybean F1 hybrids. **(A)** Enriched GO terms of genes showing additive expression in HYBSOY-1 and HYBSOY-5. **(B)** Enriched KEGG terms of genes showing additive expression in HYBSOY-1. **(C)** Enriched KEGG terms of genes showing additive expression in HYBSOY-5. Fisher’s test, *FDR<0.05 and **FDR<0.01.

For a more detailed analysis of DEGs, the genes were categorized into four major expression categories, which included 12 expression patterns (I–XII, Table 3) according to previously defined criteria [38]. In HYBSOY-1, the parental expression dominated (II, XI, IV, and IX), while transgressive down-regulation (III) had the highest proportion of the 12 expression patterns (Table 3).To gain further insights into the possible biological function of genes with nonadditive expression, we conducted KEGG and GO analysis. In HYBSOY-1 and HYBSOY-5, most genes were enriched for the GO term metabolic process as a biological process and catalytic activity as a molecular function (Fig 3A). KEGG analysis in the two F1 hybrids also showed that most genes were involved in metabolic pathway as well as the biosynthesis of secondary metabolites (Fig 3B and 3C).

**Table 3:** The 12 possible additive and nonadditive gene expression patterns in a F1 hybrid relative to its parents.

Categories	Additivity		Parental expression level dominance				Transgressive down-regulation			Transgressive up-regulation		
	I	XII	II	XI	IV	IX	III	VII	X	V	VI	VIII
Patterns	♀H♂	♀H♂	♀H♂	♀H♂	♀H♂	♀H♂	♀H♂	♀H♂	♀H♂	♀H♂	♀H♂	♀H♂
HYBSOY-1	125	0	62	0	84	0	127	0	0	3	33	3
HYBSOY-5	154	18	143	0	50	0	156	0	0	13	49	5
Common DGEs	52	0	5	0	2	0	84	0	0	0	26	0

Roman numerals represent the categorization as used by Rapp et al. (2009) with symbols for respective gene expression patterns for the maternal parent (♀), F1 hybrid (H), and paternal parent (♂).

### Additivity expression in the F1 hybrid

**Fig 4:**
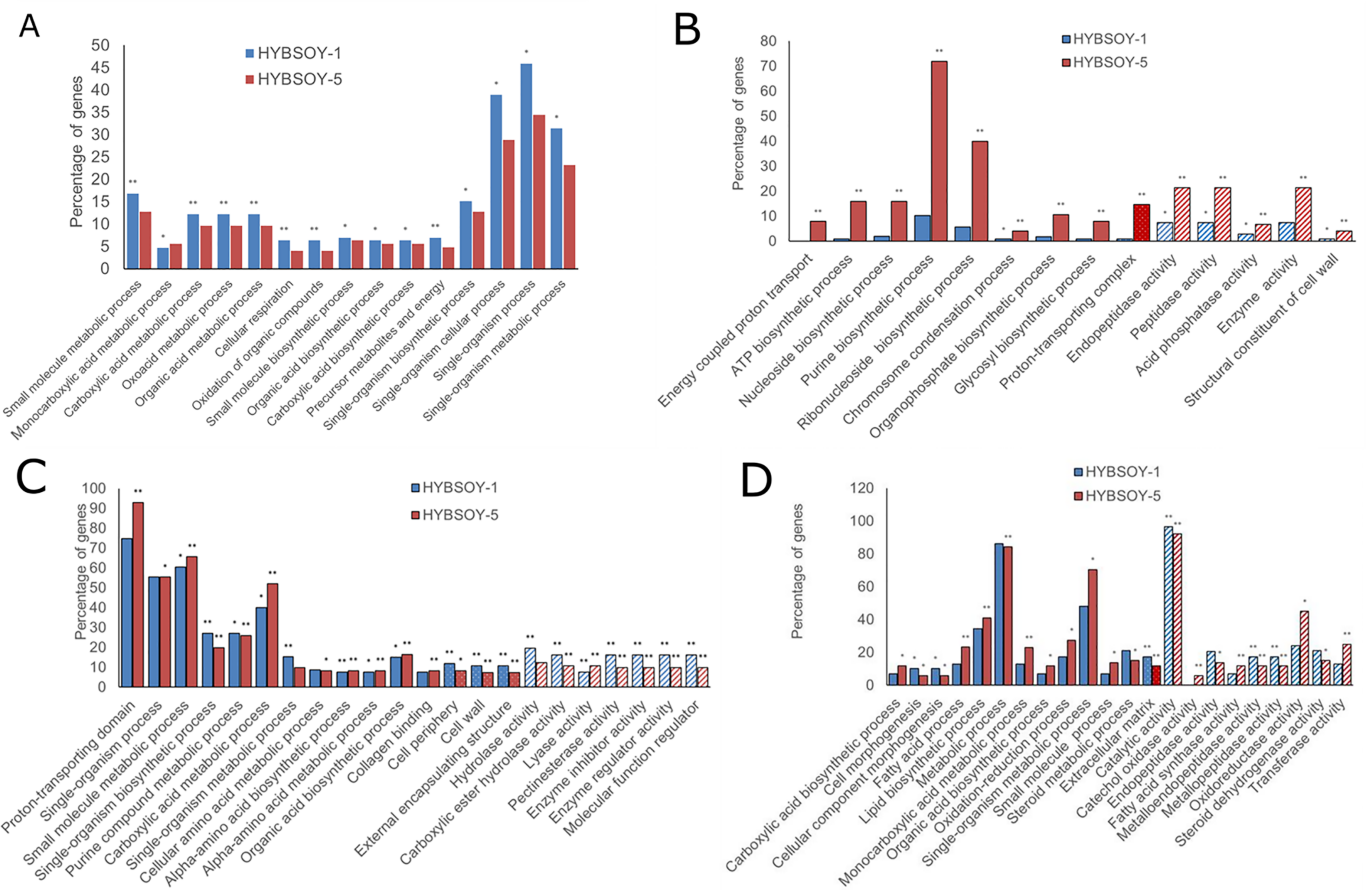
Enriched GO terms analysis in two F1 hybrids. (A) Enriched GO terms of genes showing additives expression in HYBSOY-1 and HYBSOY-5. (B) Enriched GO terms of genes showing parental expression level dominance in F1 hybrid of soybean in HYBSOY-1 and HYBSOY-5. (C) Enriched GO terms of genes showing transgressive down-regulation in HYBSOY-1. (D) Enriched GO terms of genes showing transgressive up-regulation in HYBSOY-5. Fisher’s test, *FDR<0.05 and **FDR<0.01. These GO terms were not presented without significantly enriched. Solid bar presented “biological process” GO terms; dot bar shown “cellular components” Go terms; slash bar indicated "molecular functions" GO terms.

A total of 125 and 172 DEGs with additive expression were identified in HYBSOY-1 and HYBSOY-5 (Table 3), respectively. GO classified the DEGs into differential functional groups, showing that the molecular function term and cellular component term were not significantly enriched in genes with additive expression (I + XII). The GO terms single-organism cellular process, single-organism process, and single-organism metabolic process were enriched in most biological process genes (Fig 4A).

We then classified the genes showing additive expression using KEGG analysis and found that they were significantly enriched in HYBSOY-1 for the KEGG terms metabolic pathway, photosynthesis, flavonoid pathway, and circadian rhythm, and in HYBSOY-5 for the terms starch and sucrose metabolism, pyruvate metabolism, pentose and glucoronate interconversions, flavonoid biosynthesis, and carbon fixation in photosynthetic organisms (S1A and S1B Fig).

### Parental expression level dominance in F1 hybrids

A total of 146 and 193 DEGs with parental expression level dominance were identified in HYBSOY-1 and HYBSOY-5, respectively (Table 3). GO analysis of parental expression level dominance genes (II, XI, IV, and IX) revealed large differences between that in HYBSOY-1 and in HYBSOY-5. Most HYBSOY-1 DEGs were not enriched for the GO terms that were significantly enriched in HYBSOY-5 DEGs (Fig 4B). For example, in HYBSOY-5, more than 70% DEGs were enriched for the GO term purine biosynthetic process, while 40% were enriched for the ribonucleoside biosynthetic process (Fig 4B). To classify this difference, DEGs were analyzed using KEGG pathway analysis. Most DEGs in HYBSOY-1 were enriched for metabolic pathway, while most were enriched for protein processing in the endoplasmic reticulum in HYBSOY-5 (S2C and S2D Fig). It is consistence with comparison MPH for agronomic traits of HYBSOY-1 and HYBSOY-5. MPH of measured traits in HYBSOY-5 is higher than that of HYBSOY-1, especially, four seed traits, such as NSP, HSW, and PC (Table 1). We do not know if MPH index of the metabolic compounds of HYBSOY-1 is higher than that of HYBSOY-1 since we did not measure the relative traits.

### Transgressive regulation in F1 hybrids

A total of 127 and 156 DEGs showed transgressive down-regulation in HYBSOY-1 and HYBSOY-5, respectively, while 39 and 67 DEGs showed transgressive up-regulation, respectively (Table 3). Using GO analysis and KEGG pathway analysis of transgressive regulation DEGs (III, VII, X, V, VI, and VIII), the results presented that transgressive regulation was regulated by the same functional DEGs according to (Fig 4C and 4D).

In HYBSOY-1, most DEGs were not enriched for the GO terms that were significantly enriched in HYBSOY-5 DEGs (Fig 4D). For example, in HYBSOY-5, more than 70% DEGs were enriched for the GO term purine biosynthetic process, while 40% were enriched for the term ribonucleoside biosynthetic process (Fig 4D). As before, DEGs were analyzed using KEGG pathway analysis. Most HYBSOY-1 DEGs were enriched for metabolic pathway (S2E and S2F Fig), while most were enriched for protein processing in the endoplasmic reticulum in HYBSOY-5 (S2G and S2H Fig). Comparison analysis of agronomic traits, high HPH of NNS, NPP, NSP, and HSW in HYBSOY-5 and NPP, NSP, and HSW in HYBSOY-1 were detected. HPH of NNS and NSP in HYBSOY-5 were significant higher than that in HYBSOY-1 (Table 1). These results indicated that the heterosis traits in HYBSOY-5 were regulated by transgressive genes which were enriched for protein processing.

### Heterosis genes in hybrid soybean

**Fig 5:**
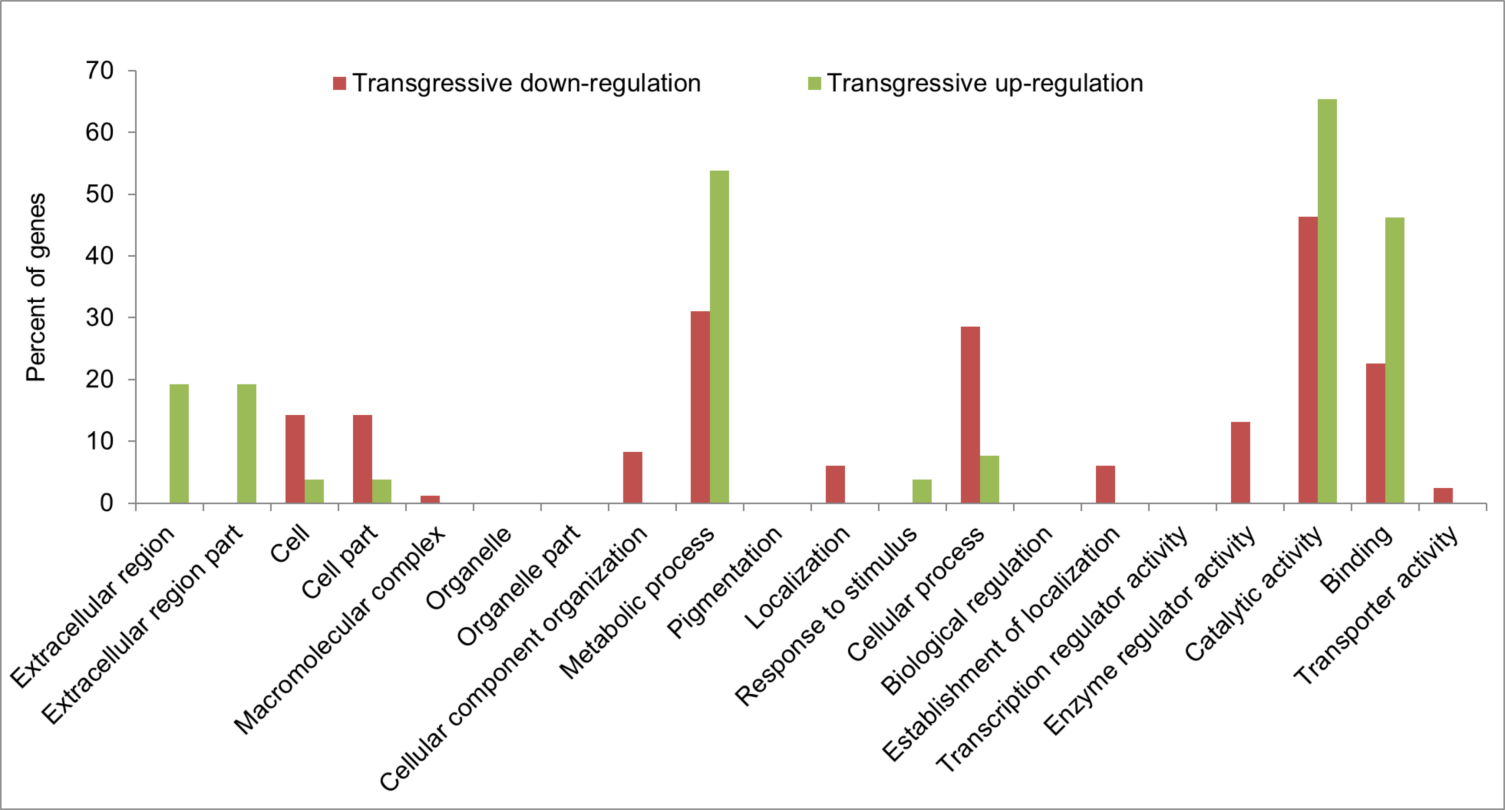
Enriched GO terms analysis of potential candidate genes involved in heterosis in F1 hybrids.

To investigate heterosis genes in soybean hybrids, DEGs common to both HYBSOY-1 and HYBSOY-5 were considered potential candidates (Table 3). A total of 169 DEGs were identified as potential heterosis genes in the two F1 hybrids. These included 52, 84, and 26 DEGs that showed additive, transgressive down-regulation (S3 Table), and transgressive up-regulation expression, respectively. DEGs with parental expression level dominance were difficult to identify in F1 hybrids. Hence, we did not find common parental expression level dominance genes from HYBSOY-1 and HYBSOY-5. Moreover, interestingly, DEGs with transgressive down- and up-regulation could be significantly identified in GO terms (Fig 5). Thus, the GO terms extracellular region and extracellular region part in the cellular component, metabolic process in the biological process, and catalytic activity and binding in molecular function in transgressive up-regulation were significantly enriched. This indicates that these genes with transgressive up-regulation were responsible for heterosis in the soybean F1 hybrids.

To identify the metabolic pathways in which the DEGs were involved in and enriched, pathway-based analysis was performed using the KEGG pathway database by KOBAS [36]. However, 84 DEGs with transgressive down-regulation cannot be significantly enriched KEGG pathway (Talbe S3), these DEGs were not performed more analysis in this study. In total, 26 DEGs with transgressive up-regulation were assigned to 11 KEGG pathways (Table 4), of which “biosynthesis of secondary metabolites” was the most representative (KO01110, 7), followed by “metalloendopeptidases” (KO01000, 5) and “ABC transporters” (KO10440, 3). Comparison analysis of MPH and HPH in two F1 hybrids revealed that these transgressive regulation genes may be potential heterosis genes in hybrid soybean.

**Table 4:** KEGG pathway enrichment of genes showing transgressive up-regulation in HYBSOY-1 and HYBSOY-5.

Gene Id	Gene Annotation	KO	Log2FC	
	**Biosynthesis of secondary metabolites**		HYBSOY-1	HYBSOY-5
Glyma.01G061100	Cytochrome P450, family 78, subfamily A, polypeptide 5	K00517	2.1092	3.7365
Glyma.09G233700	Galactosyltransferase family protein	K00734	1.3749	4.9792
Glyma.18G076900	Acyl-CoA synthetase 5	K01904	3.6822	3.3355
Glyma.08G329700	Acyl-CoA synthetase 5	K01904	5.8179	6.1658
Glyma.15G018500	Dihydroflavonol 4-reductase-like1	K13082	5.1555	5.0822
Glyma.13G355600	Dihydroflavonol 4-reductase-like1	K13082	2.7213	2.9221
Glyma.07G157200	Bifunctional dihydroflavonol 4-reductase	K13082	4.8016	4.8886
	**ABC transporters**			
Glyma.07G234400	Protein of unknown function, DUF538	K05677	6.8285	7.9232
Glyma.15G029300	Calcium-dependent phosphotriesterase superfamily protein	K10440	2.2311	2.8548
Glyma.13G345100	Calcium-dependent phosphotriesterase superfamily protein	K10440	3.0785	3.8508
	**Glycolysis / Gluconeogenesis**			
Glyma.17G075300	Aldehyde dehydrogenase 11A3	K00131	2.2145	1.663
	**Chromate reductase**			
Glyma.11G152400	NADPH:quinone oxidoreductase	K19784	2.3364	1.5053
	**Toll-like receptor signaling**			
Glyma.01G001900	Protein kinase protein with adenine nucleotide alpha hydrolases-like domain	K04733	11.166	10.453
	**Polyketide biosynthesis proteins**			
Glyma.01G073600	Chalcone and stilbene synthase family protein	K16167	11.577	11.601
	**Metalloendopeptidases**			
Glyma.02G028100	Matrixin family protein	K07761	3.0293	4.5796
Glyma.02G028200	Matrixin family protein	K07998	7.8033	9.7216
Glyma.02G028600	Matrixin family protein	K07761	4.1589	5.9359
Glyma.02G028700	Matrixin family protein	K07761	3.0786	4.3397
Glyma.02G028800	Matrixin family protein	K07761	4.0357	5.7163
	**RNA degradation**			
Glyma.14G007200	Fasciclin-like arabinogalactan family protein	K12605	4.6616	4.4344
	**Messenger RNA biogenesis**			
Glyma.15G228900	UBX domain-containing protein	K18726	6.8729	7.3827
	**DNA replication proteins**			
Glyma.15G271600	Bifunctional inhibitor/lipid-transfer protein	K02605	7.2529	8.0257
	**Transcriptional factors and others**			
Glyma.10G281800	Basic helix-loop-helix (bHLH) DNA-binding superfamily protein		3.6643	5.6741
Glyma.14G028600	Predicted AT-hook DNA-binding family protein		4.4178	4.7926
Glyma.02G285500	Predicted AT-hook DNA-binding family protein		7.3952	9.1419
Glyma.08G321400	Eukaryotic aspartyl protease family protein		1.307	2.2551

### qRT-PCR validation of transcriptome results

**Fig 6:**
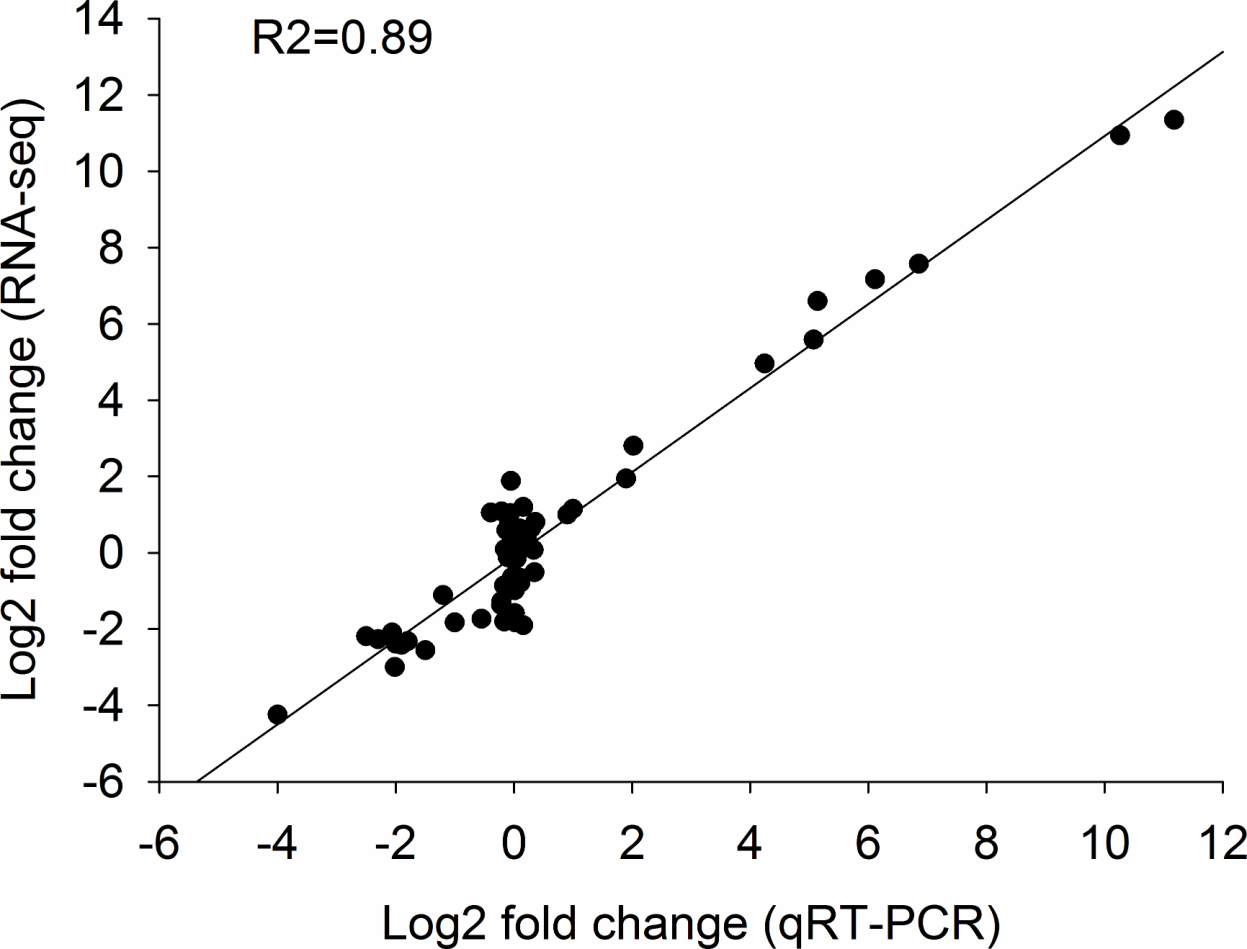
Comparison of gene expression values obtained by RNA-seq and qRT-PCR. Fold changes were calculated for 18 DEGs and a high correlation (*R*^*2*^ = 0.89) was observed between the results obtained using the two methods. The detailed information is given in S3 Fig and S1 Table.

We performed qRT-PCR to verify the results of RNA-seq analysis. We examined the expression level of twenty genes using qRT-PCR, including two non-expression genes, four high expression genes, four medium expression gene, and six low expression in five sample using RNA-sequcing. Two non-expression genes using RNA-seq analysis cannot be detected gene expression using qRT-PCR. The strong correlation (R2 = 0.89, *P*< 0.05) was observed between the expression of 18 DEGs detected by RNA-seq and qRT-PCR (Fig 6). qRT-PCR results confirmed that the reproducibility and reliability of the transcriptome data obtained in this study (S3 Fig).

## Discussion

The molecular and genetic mechanisms of heterosis are poorly understood, although differential gene expression between a hybrid and its parents is thought to be involved [9–11, 17, 39–41]. In the present study, comparative transcriptome analysis between parent plants and two F1 hybrids was conducted using next-generation sequencing (NGS) technology with no biological replicates. The transcriptome usually can be accurately analyzed using NGS technology with biological replicates. When the funding is not enough, the transcriptome analysis also was performed without biological relicates. But the gene expression profiles of key genes must be verified using qRT-PCR [42]. In our studies, 20 key genes were selected to verified the results of RNA-seq with qRT-PCR. The gene expression profiles of these genes was consistence with our transcriptome analysis. This results indicated that ourtranscriptome analysis was reliable. In present study, a total of 681 and 899 DEGs had nonadditive expression in HYBSOY-1 and HYBSOY-5, respectively. Our GO and KEGG functional analysis together indicate that these DEGs may be involved in heterosis, particularly the 26 genes with transgressive up-regulation identified in the two F1 hybrids.

### Comparative analysis of F1 hybrids

Comparative transcriptome analysis revealed a subset of DEGs that were differentially expressed between hybrids and their parents during flowering. Most DEGs were enriched in metabolic process as the biological process term and catalytic activity as the molecular function. The processes included primary metabolic, small molecular metabolic, single-organism biosynthetic process, carbohydrate metabolic process, organic acid metabolic process, and oxoacid metabolic process, which all contribute greatly to seed development. This strongly suggests that these DEGs are involved in heterosis in soybean F1 hybrids.

Comparing the DEGs of the two F1 hybrids identified those that were common to both HYBSOY-1 and HYBSOY-5. Interestingly, this did not include any DEGs with parental expression level dominance, indicating that this is not relevant to soybean heterosis. Furthermore, the GO terms extracellular region and extracellular region part in the cellular component, metabolic process in the biological process, and catalytic activity and binding in the molecular function within transgressive up-regulation were more significantly enriched than those of additive expression and transgressive down-regulation. These results suggest that genes with transgressive up-regulation are associated with heterosis in soybean F1 hybrids. A total of 26 DEGs with transgressive up-regulation were subsequently analyzed to examine the molecular mechanism of heterosis.

### The role of metabolic processes

Metabolic processes are crucial to soybean seed development because they produce fatty acids and flavonoids, and generate the seed coat. In our study, DEGs, including those with additive expression, parental expression level dominance, and transgressive regulation, were enriched in metabolic process according to GO and KEGG pathway analyses. Significant differences in metabolic process, including the biosynthesis of secondary metabolites, glycolysis/gluconeogenesis, and polyketide biosynthesis proteins, were identified in the two F1 hybrids and their parents. Four flavonoid biosynthesis pathway genes (Glyma.15G018500, Glyma.13G355600, Glyma.07G157200, and Glyma.01G073600) were identified as having transgressive up-regulation in HYBSOY-1 and HYBSOY-5. These results are consistent with the flavonoids previously reported as major metabolic compounds [19, 43, 44]. Additionally, the fatty acid biosynthesis pathway genes Glyma.18G076900 and Glyma.08G329700 were identified as having transgressive up-regulation, while Glyma.17G075300, involved in the glycolysis/gluconeogenesis pathway, was up-regulated in both F1 hybrids. These results indicate that the metabolic processes of the two F1 hybrids were significantly different compared with those of their parents, and thus may be involved in heterosis.

### The role of metalloendopeptidases

Matrix metalloproteases (MMPs) are a family of zinc-dependent endopeptidases that are widely distributed throughout all kingdoms of life. In mammals, MMPs play key roles in many physiological and pathological processes, including remodeling of the extracellular matrix [45, 46]. In plants, MMPs is likely to play a role in plant extracellular cell matrix degradation [47]. *Arabidopsis thaliana* encodes five MMP-like proteins (At-MMPs), which may be involved in different proteolytic processes during plant growth and development [48]. More ever, MMPs have been reported that they are involved in rice heterosis [49–51]. In our study, five MMPs (Glyma.02G028100, Glyma.02G028200, Glyma.02G028600, Glyma.02G028700, and Glyma.02G028800) were identified to have transgressive up-regulation with a significant fold-change range from 3.0 to 10.0 (Table 4) in both HYBSOY-1 and HYBSOY-5. These results indicate that MMPs may be involved in soybean seed development and heterosis in F1 hybrids.

### ABC transporter and other DEGs in heterosis

Three ABC transporters (Glyma.07G234400, Glyma.15G029300, and Glyma.13G345100) were identified to have significant differences in expression between F1 hybrids and their parents. Typically, ABC transporters transport ligands across cellular lipid membranes, and are involved in the uptake of nutrients and elimination of waste products, energy generation, and cell signaling [52]. In soybean, ABC transporters were shown to play a role in seed development [53, 54]. Our results indicate that ABC transporters are involved in heterosis regulation in soybean hybrid lines. Moreover, genes involved in RNA degradation (Glyma.14G007200), mRNA biogenesis (Glyma.15G228900), DNA replication (Glyma.15G271600), transcriptional factors (Glyma.10G281800), and DNA binding (Glyma.14G028600 and Glyma02G285500) were identified as enriched in transgressive up-regulation in both HYBSOY-1 and HYBWOY-5. These results together suggest that the heterosis of soybean F1 hybrids is controlled by genes of several different pathways.

## Supporting information

S1 Fig Pearson correlation analysis among samples. If R^2^ between two samples <0.8, it reflects a poor quality of RNA-seq.
(DOCX)

S2 Fig Enriched KEGG terms analysis in two F1 soybean hybrids. **(A)** Enriched KEGG terms of genes showing additive expression in HYBSOY-1. **(B)** Enriched KEGG terms of genes showing additive expression in HYBSOY-5. **(C)** Enriched KEGG terms of genes showing parental expression in HYBSOY-1. **(D)** Enriched KEGG terms of genes showing parental expression in HYBSOY-5. **(E)** Enriched KEGG terms of genes showing transgressive down-regulation in HYBSOY-1. **(F)** Enriched KEGG terms of genes showing transgressive down-regulation in HYBSOY-5. **(G)** Enriched KEGG terms of genes showing transgressive up-regulation in HYBSOY-1. **(H)** Enriched KEGG terms of genes showing transgressive up-regulation in HYBSOY-5. Fisher’s test,*FDR<0.05 and **FDR<0.01.
(DOCX)

S3 Fig qRT-PCR verification of DEGs. (DOCX)

S1 Table Primer sequences for qRT-PCR validation of RNA-Seq. (DOCX)

S2 Table Statistics of gene expression levels. (DOCX)

S3 Table Transgressive down-regulation genes in HYBSOY-1 and HYBSOY-5. (DOCX)
