# Supplementary material for: Comparative transcriptome analysis of flower heterosis in two soybean F1 hybrids by RNA-seq
Source: PLoS One. 2017 Jul 14;12(7):e0181061. doi: 10.1371/journal.pone.0181061 (PMC5510844; doi:10.1371/journal.pone.0181061)
Supplement: S1 Fig — If R2 between two samples <0.8, it reflects a poor quality of RNA-seq. (DOCX) [file pone.0181061.s001.docx]

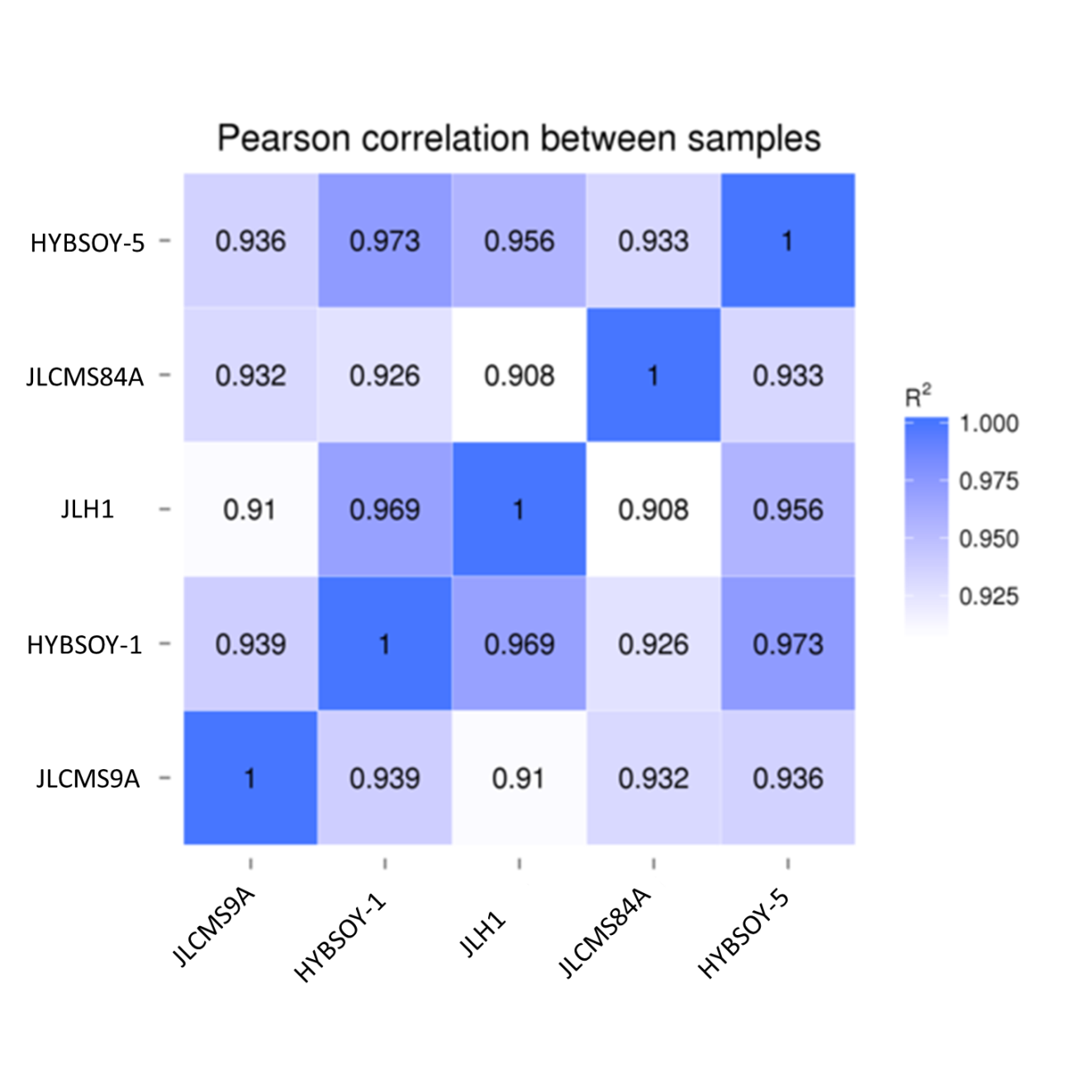


S1 Fig. Pearson correlation analysis among five samples. If R^2^ less than 0.8 between two sample, it presents that the difference among of samples is significant.
