# Supplementary material for: Comparative transcriptome analysis of flower heterosis in two soybean F1 hybrids by RNA-seq
Source: PLoS One. 2017 Jul 14;12(7):e0181061. doi: 10.1371/journal.pone.0181061 (PMC5510844; doi:10.1371/journal.pone.0181061)
Supplement: S2 Fig — (A) Enriched KEGG terms of genes showing additive expression in HYBSOY-1. (B) Enriched KEGG terms of genes showing additive expression in HYBSOY-5. (C) Enriched KEGG terms of genes showing parental expression in HYBSOY-1. (D) Enriched KEGG terms of genes showing parental expression in HYBSOY-5. (E) Enriched KEGG terms of genes showing transgressive down-regulation in HYBSOY-1. (F) Enriched KEGG terms of genes showing transgressive down-regulation in HYBSOY-5. (G) Enriched KEGG terms of genes showing transgressive up-regulation in HYBSOY-1. (H) Enriched KEGG terms of genes showing transgressive up-regulation in HYBSOY-5. Fisher’s test,*FDR<0.05 and **FDR<0.01. (DOCX) [file pone.0181061.s002.docx]

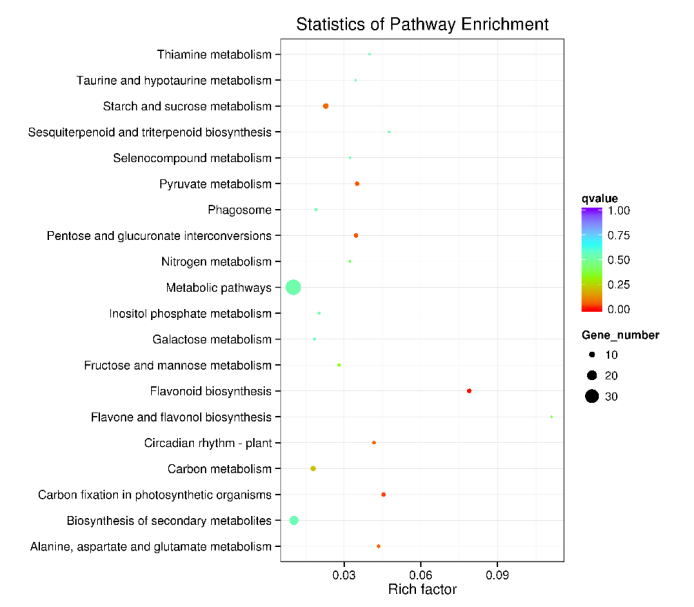

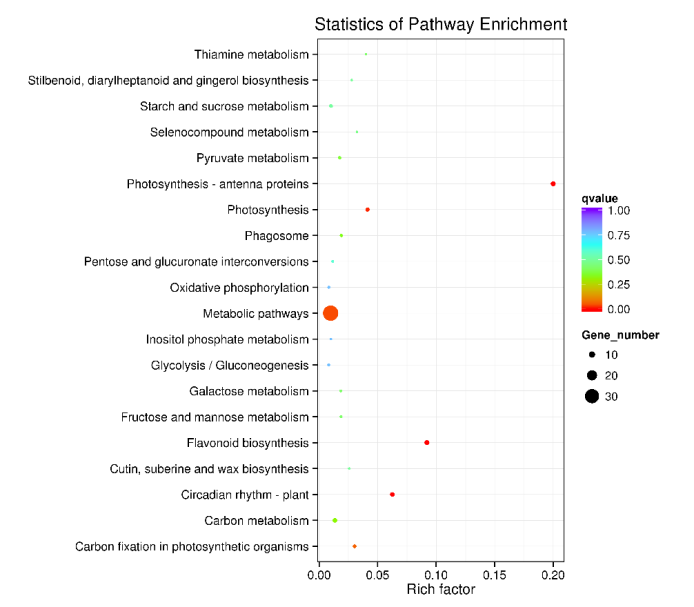


B

A


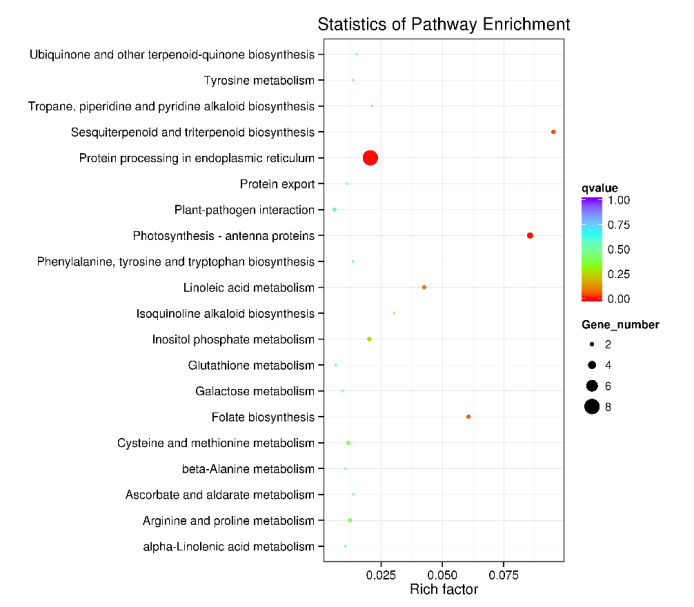

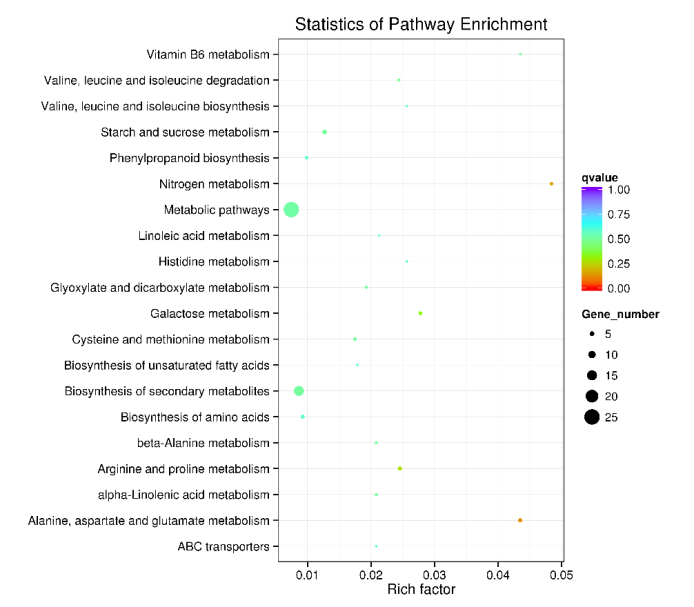


D

C


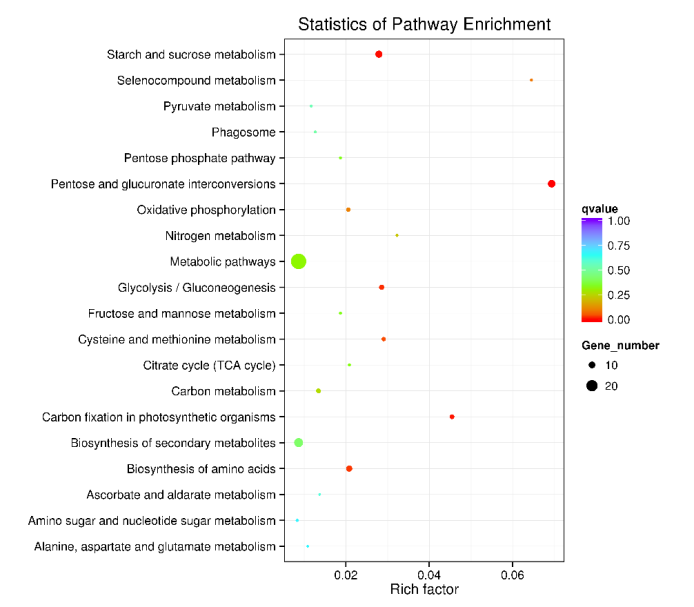

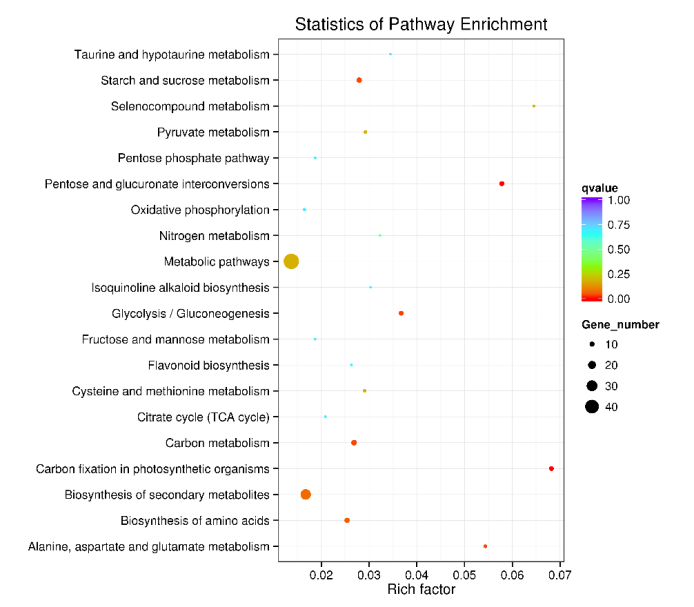


H

G

F

E


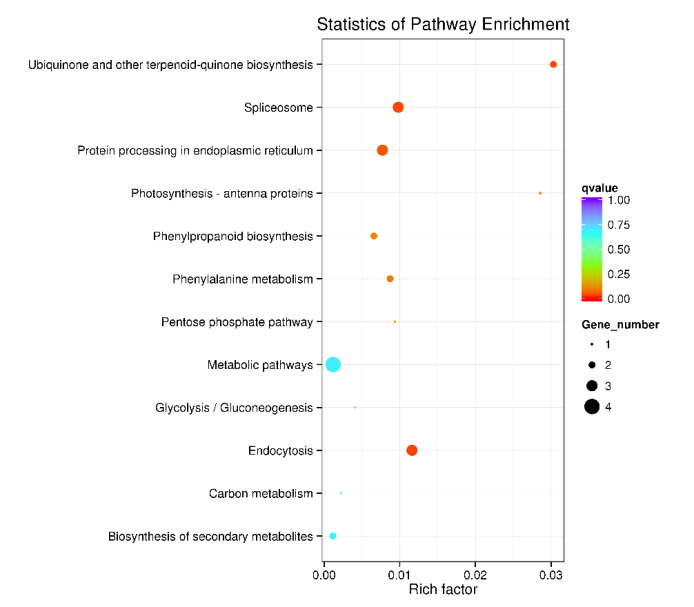

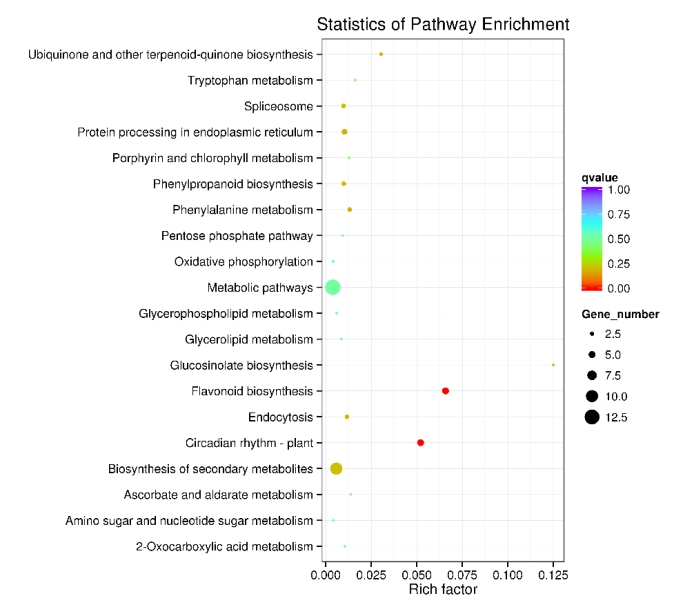


S2 Fig. Enriched KEGG terms analysis in two F1 soybean hybrids. (A) Enriched KEGG terms of genes showing additivity expression genes in HYBSOY-1. (B) Enriched KEGG terms of genes showing additivity expression genes in HYBSOY-5. (C) Enriched KEGG terms of genes showing Paternal expression genes in HYBSOY-1. (D) Enriched KEGG terms of genes showing Paternal expression genes in HYBSOY-5. (E) Enriched KEGG terms of genes showing transgressive down-regulation genes in HYBSOY-1. (F) Enriched KEGG terms of genes showing transgressive down-regulation genes in HYBSOY-5. (G) Enriched KEGG terms of genes showing transgressive up-regulation genes in HYBSOY-1. (H) Enriched KEGG terms of genes showing transgressive up-regulation genes in HYBSOY-5. Fisher test,*FDR<0.05 and **FDR<0.01.
