# Supplementary material for: Comparative transcriptome analysis of flower heterosis in two soybean F1 hybrids by RNA-seq
Source: PLoS One. 2017 Jul 14;12(7):e0181061. doi: 10.1371/journal.pone.0181061 (PMC5510844; doi:10.1371/journal.pone.0181061)
Supplement: S3 Fig — (DOCX) [file pone.0181061.s003.docx]

S3 Fig. qRT-PCR verification of DEGs. The qRT-PCR primer sequences were shown in Table S1. ABCT, ACT11, and CONS4 were used as reference gene. The relative gene expression was calculated based on the description of Methods.
