# Supplementary material for: Comparative transcriptome analysis of flower heterosis in two soybean F1 hybrids by RNA-seq
Source: PLoS One. 2017 Jul 14;12(7):e0181061. doi: 10.1371/journal.pone.0181061 (PMC5510844; doi:10.1371/journal.pone.0181061)
Supplement: S1 Table — (DOCX) [file pone.0181061.s004.docx]

S1 Table.Primer sequences for qRT-PCR validation of RNA-Seq.

| Gene ID | Sequence |
| --- | --- |
| Glyma.01G001900_F | CATTGTACGAGGATCAGGACAG |
| Glyma.01G001900_R | GCGATTGTGAAGAGTAGGAAGA |
| Glyma.01G061100_F | GGGTGTTATTGAGGAGCATAGA |
| Glyma.01G061100_R | GCTTGTTCTCGTTCTCCAAATC |
| Glyma.01G073600_F | GTCGCGTAATGCTCTACTTTCT |
| Glyma.01G073600_R | CCAGGGTTGTTCTCTGCTATG |
| Glyma.17G217900_F | TTGCTCCAGGGAGAGGATTA |
| Glyma.17G217900_R | GGAAATAGTTGGTCCTTGCTTTG |
| Glyma.13G024200_F | CCGACCTTGGTAGTGGTATTTC |
| Glyma.13G024200_R | GGGAAAGAAGACCCTGTTGAG |
| Glyma.10G214500_F | CAGATCAACATTGTGGTGGTTG |
| Glyma.10G214500_R | ATGCCCTGGAGCTTCTTTC |
| Glyma.13G020700_F | CTGTGGTTTCGCTGGATAGTAG |
| Glyma.13G020700_R | GTAAACGGCGGGAGTAACTATG |
| Glyma.13G012400_F | CCTCCAATGGATCCTCGTTAAG |
| Glyma.13G012400_R | CGTCGTGAGACAGGTTAGTTT |
| Glyma.03G191600_F | TGAAGACAAAGCAAGGAAGGA |
| Glyma.03G191600_R | CCGTACCTGGGAAAGAGTAAAG |
| Glyma.02G028100_F | AACATCGGCTGGTTACTTCC |
| Glyma.02G028100_R | CAAGTCCAAGTAGATGCCCTATC |
| Glyma.02G028200_F | CCTTATCCTTCTCCACCAATCC |
| Glyma.02G028200_R | CGCTCGGGAGCTTCTTTATATC |
| Glyma.02G028600_F | TAACATGGGCTGGTTACTTCC |
| Glyma.02G028600_R | CAAGTCCAAGTAGATGCCCTATC |
| Glyma.02G028700_F | GGTGGTACTTATGGACGGTAAC |
| Glyma.02G028700_R | CAAGTCCAAGTAGATGCCCTATC |
| Glyma.02G028800_F | CGGCGTAGAACTTGCAATAGA |
| Glyma.02G028800_R | GACGACAGACAGGCCTTTAAT |
| Glyma.02G285500_F | GCAACGTGGCCTTTACATATTC |
| Glyma.02G285500_R | AATCTCGAATCGACCGTGAAG |
| Glyma.07G157200_F | AGACATCTGGCAGGCTTATTT |
| Glyma.07G157200_R | TGGCTGCTGCACCTATTT |
| Glyma.07G234400_F | GTGTGGAAGGGTTAGAGAAACT |
| Glyma.07G234400_R | TGTGACCTCCACAGCATAAC |
| Glyma.08G321400_F | CGGCATTTCGGCAGTTTATG |
| Glyma.08G321400_R | CCTCGGAACTGAAATCTCCTTAT |
| Glyma.08G329700_F | GATCTTTGTGCCATGCCATTC |
| Glyma.08G329700_R | GGTCACACCAAAGAGTGTAGAG |
| Glyma.09G233700_F | CACAAGGACTTCCTCAGGTTAG |
| Glyma.09G233700_R | AGCATCCCACGTAGAAATGATAG |
| ABCT_F | CTTTGCTTTTATTCCGAATGG |
| ABCT_R | GCCTGCTTCAGATAAAATAGAT |
| ACT11-F | CGGTGGTTCTATCTTGGCATC |
| ACT11-R | GTCTTTCGCTTCAATAACCCTA |
| CONS4-F | GATCAGCAATTATGCACAACG |
| CONS4-R | CCGCCACCATTCAGATTATGT |
