# Supplementary material for: Comparative transcriptome analysis of flower heterosis in two soybean F1 hybrids by RNA-seq
Source: PLoS One. 2017 Jul 14;12(7):e0181061. doi: 10.1371/journal.pone.0181061 (PMC5510844; doi:10.1371/journal.pone.0181061)
Supplement: S2 Table — (DOCX) [file pone.0181061.s005.docx]

S2 Table. Statistics of gene expression level.

| FPKM^a^ Interval | JLCMS9A | HYBSOY-1 | JLH1 | JLCMS84A | HYBSOY-5 |
| --- | --- | --- | --- | --- | --- |
| 0~1 | 20412(36.42%) | 21458(38.29%) | 21153(37.74%) | 23860(42.57%) | 21539(38.43%) |
| 1~3 | 7949(14.18%) | 8648(15.43%) | 8512(15.19%) | 9182(16.38%) | 8635(15.41%) |
| 3~15 | 17295(30.86%) | 17052(30.43%) | 17215(30.72%) | 16115(28.75%) | 17123(30.55%) |
| 15~60 | 7825(13.96%) | 6604(11.78%) | 6757(12.06%) | 5031(8.98%) | 6463(11.53%) |
| >60 | 2563(4.57%) | 2282(4.07%) | 2407(4.29%) | 1856(3.31%) | 2284(4.08%) |

^a^FPKM present expected number of Fragments Per Kilobase of transcript sequence per Millions base pairs sequenced.
