# Supplementary material for: Comparative transcriptome analysis of flower heterosis in two soybean F1 hybrids by RNA-seq
Source: PLoS One. 2017 Jul 14;12(7):e0181061. doi: 10.1371/journal.pone.0181061 (PMC5510844; doi:10.1371/journal.pone.0181061)
Supplement: S3 Table — (DOCX) [file pone.0181061.s006.docx]

S3 Table. Transgressive down-regulation genes in HYBSOY-1 and HYBSOY-5.

| Gene ID | Gene Annotation | Log2FC |  |
| --- | --- | --- | --- |
|  |  | HYBSOY-1 | HYBSOY-5 |
| Glyma.01G097400 | phosphoenolpyruvate carboxykinase 1 | -5.0905 | -6.6925 |
| Glyma.01G111400 | Plant invertase/pectin methylesterase inhibitor superfamily | -12.776 | -6.8938 |
| Glyma.01G118000 | Thiamine pyrophosphate dependent pyruvate decarboxylase family protein | -5.8588 | -4.2347 |
| Glyma.01G184500 | Transducin/WD40 repeat-like superfamily protein | -3.7016 | -4.2401 |
| Glyma.02G011500 | Plant invertase/pectin methylesterase inhibitor superfamily protein | -2.7016 | -8.6034 |
| Glyma.02G052000 | S-adenosyl-L-methionine-dependent methyltransferases superfamily protein | -4.0826 | -6.2227 |
| Glyma.02G056000 |  | -3.1095 | -3.9353 |
| Glyma.02G154400 | glutathione S-transferase 7 | -8.2652 | -5.3451 |
| Glyma.02G180200 |  | -5.3767 | -7.1087 |
| Glyma.02G266500 |  | -9.1592 | -5.0446 |
| Glyma.03G021400 | WD-40 repeat family protein / beige-related | -5.9784 | -3.7504 |
| Glyma.03G133600 | alpha carbonic anhydrase 4 | -5.7398 | -5.7847 |
| Glyma.03G193400 | Subtilisin-like serine endopeptidase family protein | -10.557 | -8.9868 |
| Glyma.03G215700 | Plant invertase/pectin methylesterase inhibitor superfamily protein | -5.3728 | -5.0331 |
| Glyma.03G223400 | Plant invertase/pectin methylesterase inhibitor superfamily | -9.07 | -13.223 |
| Glyma.04G003900 | glycosyl hydrolase family 35 protein | -12.123 | -10.723 |
| Glyma.04G005300 | rotamase CYP 3 | -3.995 | -2.5253 |
| Glyma.04G022800 | SGNH hydrolase-type esterase superfamily protein | -11.115 | -5.048 |
| Glyma.05G041400 | plant intracellular ras group-related LRR 1 | -4.8879 | -3.9623 |
| Glyma.05G073100 | glyceraldehyde-3-phosphate dehydrogenase C2 | -4.7059 | -6.6185 |
| Glyma.05G090100 | Cobalamin-independent synthase family protein | -5.5955 | -5.3422 |
| Glyma.06G005100 | rotamase CYP 3 | -2.8488 | -3.9158 |
| Glyma.06G125300 | arabinogalactan protein 16 | -5.0543 | -7.1464 |
| Glyma.07G024200 | Plant invertase/pectin methylesterase inhibitor superfamily | -8.3344 | -7.3202 |
| Glyma.07G024600 | Plant invertase/pectin methylesterase inhibitor superfamily | -8.114 | -7.0372 |
| Glyma.07G025900 | H(+)-ATPase 9 | -4.0382 | -2.9445 |
| Glyma.07G026600 | Plant invertase/pectin methylesterase inhibitor superfamily | -8.1062 | -11.361 |
| Glyma.07G032400 | Late embryogenesis abundant protein (LEA) family protein | -4.3781 | -6.423 |
| Glyma.07G125700 | profilin 4 | -4.2036 | -5.6696 |
| Glyma.07G148000 | Carbohydrate-binding-like fold | -11.217 | -5.2786 |
| Glyma.07G198100 | ralf-like 19 | -4.5945 | -7.4054 |
| Glyma.07G225300 | SKU5 similar 12 | -4.4548 | -7.4796 |
| Glyma.07G225400 | SKU5 similar 12 | -5.0978 | -8.6216 |
| Glyma.08G008900 | phosphate transporter 3;1 | -3.2727 | -5.1315 |
| Glyma.08G255700 | Late embryogenesis abundant protein (LEA) family protein | -5.7212 | -7.7899 |
| Glyma.08G255900 | Methylenetetrahydrofolate reductase family protein | -6.3427 | -7.3702 |
| Glyma.09G021900 | Plant invertase/pectin methylesterase inhibitor superfamily protein | -6.0088 | -7.6158 |
| Glyma.09G284200 | Major facilitator superfamily protein | -6.6485 | -5.2135 |
| Glyma.09G284600 | Major facilitator superfamily protein | -6.5856 | -5.1502 |
| Glyma.10G036800 | phosphate transporter 1;4 | -6.7852 | -7.2526 |
| Glyma.10G054500 | methionine adenosyltransferase 3 | -3.5544 | -4.9905 |
| Glyma.10G066700 | Aldolase superfamily protein | -3.1653 | -3.2697 |
| Glyma.10G094600 |  | -5.2052 | -3.8779 |
| Glyma.10G130600 | Late embryogenesis abundant protein (LEA) family protein | -4.4179 | -9.8985 |
| Glyma.10G223100 |  | -4.7992 | -8.486 |
| Glyma.10G246600 |  | -3.281 | -6.5924 |
| Glyma.11G107600 | glycosyl hydrolase family 35 protein | -3.8445 | -4.6181 |
| Glyma.11G254700 | S-adenosyl-L-homocysteine hydrolase | -2.5604 | -1.9787 |
| Glyma.11G255400 | Protein of unknown function (DUF803) | -11.663 | -5.7222 |
| Glyma.12G032600 | glycosyl hydrolase family 35 protein | -4.6118 | -7.0487 |
| Glyma.12G040300 | vacuolar H+-ATPase subunit E isoform 2 | -5.0203 | -7.4167 |
| Glyma.12G206100 |  | -9.9831 | -4.9249 |
| Glyma.13G064700 | Pectate lyase family protein | -4.9404 | -6.7255 |
| Glyma.13G141600 | methionine adenosyltransferase 3 | -5.2599 | -6.3358 |
| Glyma.13G151800 | Aldolase superfamily protein | -3.2119 | -3.2806 |
| Glyma.13G178300 | ralf-like 19 | -5.8554 | -10.361 |
| Glyma.13G203400 | ADP/ATP carrier 2 | -4.6913 | -6.3237 |
| Glyma.13G230300 | Pollen Ole e 1 allergen and extensin family protein | -8.3336 | -9.6519 |
| Glyma.14G051100 |  | -5.0384 | -7.4858 |
| Glyma.14G072500 | ADPGLC-PPase large subunit | -6.1165 | -6.0331 |
| Glyma.14G093300 | NADH-ubiquinone oxidoreductase 20 kDa subunit, mitochondrial | -4.2565 | -8.9497 |
| Glyma.14G133200 | FASCICLIN-like arabinogalactan protein 3 precursor | -5.6513 | -6.7459 |
| Glyma.15G004300 | H(+)-ATPase 9 | -7.6434 | -8.1875 |
| Glyma.15G082300 | Pollen Ole e 1 allergen and extensin family protein | -6.7538 | -7.4686 |
| Glyma.15G142900 | Mitochondrial import inner membrane translocase subunit Tim17/Tim22/Tim23 family protein | -4.3507 | -4.4667 |
| Glyma.15G183700 | Plant invertase/pectin methylesterase inhibitor superfamily | -6.9869 | -6.7481 |
| Glyma.15G202600 |  | -1.4241 | -2.4877 |
| Glyma.17G027300 | NAD(P)-binding Rossmann-fold superfamily protein | -3.7685 | -3.6915 |
| Glyma.17G184900 | Cobalamin-independent synthase family protein | -8.6061 | -5.319 |
| Glyma.17G238200 | N-terminal nucleophile aminohydrolases (Ntn hydrolases) superfamily protein | -3.5058 | -1.5395 |
| Glyma.17G252500 | ADPGLC-PPase large subunit | -5.3714 | -4.8051 |
| Glyma.18G240000 | Late embryogenesis abundant protein (LEA) family protein | -8.8494 | -6.523 |
| Glyma.18G278600 | Late embryogenesis abundant protein (LEA) family protein | -4.1306 | -6.2924 |
| Glyma.18G279300 | Late embryogenesis abundant protein (LEA) family protein | -5.3915 | -9.69 |
| Glyma.18G289600 | heat shock cognate protein 70-1 | -4.1811 | -5.634 |
| Glyma.19G033800 | reversibly glycosylated polypeptide 2 | -4.3167 | -4.002 |
| Glyma.19G078300 | glyceraldehyde-3-phosphate dehydrogenase C2 | -7.0812 | -9.0107 |
| Glyma.19G212200 | Plant invertase/pectin methylesterase inhibitor superfamily protein | -6.8933 | -8.6648 |
| Glyma.19G220600 | Plant invertase/pectin methylesterase inhibitor superfamily | -4.603 | -6.9076 |
| Glyma.19G245800 | Malectin/receptor-like protein kinase family protein | -4.2812 | -6.8939 |
| Glyma.20G025200 | SKU5 similar 12 | -5.2931 | -8.1605 |
| Glyma.20G081400 | Late embryogenesis abundant protein (LEA) family protein | -7.4611 | -5.8159 |
| Glyma.20G146700 |  | -3.0033 | -9.1572 |
| Glyma.20G218100 | UDP-Glycosyltransferase superfamily protein | -3.1823 | -2.8695 |
